# Cryopreservation of Natural Killer Cells Pre-Complexed with Innate Cell Engagers

**DOI:** 10.3390/antib11010012

**Published:** 2022-02-09

**Authors:** Uwe Reusch, Kristina Ellwanger, Ivica Fucek, Thomas Müller, Ute Schniegler-Mattox, Joachim Koch, Michael Tesar

**Affiliations:** Affimed GmbH, 69120 Heidelberg, Germany; u.reusch@affimed.com (U.R.); k.ellwanger@affimed.com (K.E.); i.fucek@affimed.com (I.F.); t.mueller@affimed.com (T.M.); u.schniegler-Mattox@affimed.com (U.S.-M.); j.koch@affimed.com (J.K.)

**Keywords:** adoptive transfer, bispecific antibody, cryopreservation, innate cell engager, innate immune cells, natural killer cell

## Abstract

Innate cell engager (ICE^®^) constructs are bispecific tetravalent antibodies targeting specific tumor antigens and simultaneously engaging natural killer (NK) cell and macrophage receptors for the destruction of tumor cells. Pre-complexing of ICE^®^ constructs with adoptive NK cells is a novel approach to enhance NK cell activity. The suitability of such complexes for cryopreservation, whilst retaining the biological activity and specificity, may enable the development of off-the-shelf NK cell products. This study investigates the binding affinity of ICE^®^ constructs targeting EpCAM and NK cell receptors CD16A, NKG2D, or NKp46 to the corresponding antigens, the ICE^®^ antitumor activity, and feasibility of cryopreservation. Cell surface retention assays using primary NK cells confirmed a substantially slower ICE^®^ construct dissociation kinetics compared with control molecules, suggesting the formation of durable complexes independently of the CD16A polymorphism. The high-affinity NK cell and EpCAM/CD16A ICE^®^ complexes were superior to those engaging NKG2D or NKp46 receptors when tested for the NK-cell-mediated elimination of EpCAM-expressing tumor cells. Moreover, the potency and efficacy of these complexes were unaffected after a single freeze–thaw cycle. CD16A-selective ICE^®^ drug candidates complexed with NK cells hold promise as novel cryopreserved off-the-shelf NK cell products with chimeric antigen receptor-like NK cell properties, capable of effective depletion of tumor cells.

## 1. Introduction

The innate immune system is a prime surveyor of and responder to an invasion by foreign antigens and infections, and to the tumorigenic transformation of normal tissue, and provides the first line of defense against pathogens and tumorigenesis [1,2]. Natural killer (NK) cells and macrophages are critical components of the innate immune system acting in tumor immunosurveillance, leading to the destruction of transformed cells via the antibody-dependent cellular cytotoxicity (ADCC) and antibody-dependent cellular phagocytosis (ADCP) mechanisms, respectively [3,4].

Beyond the tumor immunosurveillance stage, NK cells and macrophages play a significant role in facilitating the elimination of tumor cells in cancers that have already progressed [3,4]. High levels of tumor-infiltrating NK cells associate with an improved prognosis in patients with some cancers, including those with breast, head and neck, squamous cell lung, and prostate cancer, and gastrointestinal stromal tumors, and neurofibroblastoma. Whilst, in patients with hepatocellular carcinoma, a higher intratumoral density of NK cells correlates with longer overall survival and disease-free progression [5]. 

NK cell activity in tumor tissue depends on the tumor cell-surface ligand repertoire, which mediates either the activation or inhibition of NK cells via the corresponding receptors. NKp46, a type I transmembrane receptor, is expressed on nearly all resting human NK cells [6] and it is a major receptor involved in triggering the activation of NK cells in response to abnormal cells expressing insufficient amounts of the human leukocyte antigen (HLA)-class I molecules [7]. NKG2D, a type II transmembrane receptor for the major histocompatibility complex (MIC) A, MICB and UL16 binding proteins (ULBPs), activates NK cells in response to infections and oncogenic transformation [6]. NKG2D expression is not limited to NK cells and can also be found on T cells, where it exerts diverse functions, and more recently, the expression of NKG2D has also been identified on B cells [8]. CD16A is among the strongest NK cell-activating receptors and the main Fc receptor expressed on NK cells that, upon engagement, triggers cytotoxic effects against cancer cells. Human CD16A is also expressed on macrophages and some circulating monocytes [9].

Significant advances have been made in evaluating novel therapeutic approaches that harness innate immune cells for targeting tumor cells. The adoptive transfer of NK cells has shown efficacy in preclinical models of ovarian cancer, glioblastoma, and metastatic colorectal cancer and acute myeloid leukemia, but its clinical efficacy is still limited. However, a favorable safety profile of adoptively transferred NK cells has been demonstrated in Phase 1 and some pilot studies [10,11,12,13]. 

NK cells can be genetically modified to express different chimeric antigen receptors (CAR) specific for cell-surface antigens in cancer cells. Currently, 20 ongoing trials are investigating CAR-NK cells for the treatment of patients with hematological malignancies and solid tumors [14]. CAR-NK cells targeting CD19-expressing B-cell malignancies showed a clinical response in 73% (8/11) of patients, and 88% of these patients (7/8) had a complete response [15]. CAR-NK cell therapy was well tolerated, without the development of cytokine release syndrome, neurotoxicity, or graft-versus-host disease [15]. However, the major drawbacks of CAR-NK cell therapy are the laborious and time-consuming process of CAR-NK cell engineering that often predisposes NK cells to apoptosis and low exogenous gene expression levels, the lack of CAR-NK cell persistence in the body, and the loss of cell viability and activity when exposed to freeze–thaw cycles [16,17,18]. 

Bi- and tri-specific killer engagers (BiKEs and TriKEs) have emerged as a new therapeutic entity designed to stabilize, activate and redirect NK cells to cancer cells expressing specific antigens. BiKEs and TriKEs harbor two and three, respectively, single chain antibody variable fragments specific to distinct cancer antigens and CD16 or/and other NK-cell receptors, such as NKp46 or NKp30. These therapeutic agents have been shown to induce ADCC and are currently undergoing preclinical and early phase clinical evaluation [19,20,21,22,23]. 

Recently, an innovative fit-for-purpose redirected optimized cell killing (ROCK^®^) platform has been developed to generate an array of bispecific tetravalent CD16A-binding antibodies, the so-called innate cell engager (ICE^®^, Affimed GmbH, Heidelberg, Germany) constructs, aiming to maximize the potential of NK cells and macrophages in targeting cancer cells [24]. Its unique modular nature enables the design of constructs with the specificity to distinct cancer antigens and the CD16A or other NK-cell receptors, leading to NK cell and macrophage recruitment, independently of the CD16A 158V/F polymorphism, and the activation of NK cell-mediated ADCC and macrophage-mediated ADCP [24]. One of the most advanced ICE^®^ constructs undergoing clinical evaluation is AFM13, a CD30-targeting ICE^®^. Patients with relapsed or refractory (R/R) Hodgkin lymphoma (HL) treated with AFM13 reported mild-to-moderate adverse events, and 11.5% (3/26) of treated and evaluable patients achieved partial remission, whilst 50% (13/26) had stable disease [25]. In heavily pretreated patients (*n* = 14) with relapsed or refractory (R/R) CD30-expressing lymphoma with cutaneous involvement, AFM13 was well tolerated and the objective response rate was 40% [26]. 

It has, however, been reported that in certain cancers, such as primary colorectal, non-small cell lung and liver tumors, NK cell density is reduced [27,28,29]. Furthermore, the tumor microenvironment may produce immunosuppressive molecules to partially compromise NK cell functions [5]. To overcome these potential hurdles and to maximize the activity of ICE^®^ constructs, pre-complexing of ICE^®^ constructs with NK cells presents a promising therapeutic option. In preclinical models, the combination of AFM13 and adoptive NK cells showed an enhanced NK cell activity and cytotoxicity against CD30-expressing lymphoma cells compared with the conventional NK cell treatment [30]. Therefore, the development of a methodology capable of producing off-the-shelf NK cell products will be essential for such a strategy to be successful in terms of bringing down the manufacturing costs and time to ensure that these therapies are delivered to patients in the most effective way. 

This study investigated the suitability for cryopreservation of NK cells pre-complexed with ICE^®^ constructs and the ability of such complexes to maintain viability, specificity, and cytotoxicity against cancer cells upon their revival from the frozen state.

## 2. Material and Methods

### 2.1. Generation of Recombinant Antibodies, Soluble Antigens and Cell-Surface Antigens

Recombinant antibodies and antigens were generated as previously described [24]. Gene sequences for different recombinant proteins (antigens, antibodies, and controls) were synthesized by GeneART Gene Synthesis (Thermo Fisher Scientific, Regensburg, Germany) or derived by polymerase chain reaction (PCR). The expression vectors, encoding these recombinant proteins, were generated by cloning the respective sequence elements into the mammalian expression vector pcDNA5/FRT (Thermo Fisher Scientific, Waltham, MA, USA) or a modified version thereof using standard molecular biology techniques.

Soluble recombinant antigen variants were constructed as fusion proteins comprising the extracellular domain (ECD) of EpCAM (Uniprot: P16422), NKG2D (Uniprot: P26718), NKp46 (Uniprot: O76036), CD16A (Uniprot: P08637, *F* *→ V*: VAR_003960), CD16B (Uniprot: O75015), the monomeric human IgG1 Fc with the effector function silenced by the introduction of the L234F, L235E, and D265A mutations, and Avi-Tag [31]. For the expression of cell-surface-anchored antigen variants, ECD sequences were either fused to the transmembrane domain (TMD) of human epidermal growth factor receptor [32], anchored via glycosylphosphatidylinositol (GPI), using the human CD16B endogenous sequence with the full-length propeptide sequence for post-translational processing and lipidation for GPI-anchorage, or the endogenous full-length sequence of the selected antigen, containing the ECD, TMD and intracellular domain, was used. NKG2D was co-expressed with full length human DAP10 (Uniprot: Q9UBK5). For that purpose, the original vector was modified to contain two cytomegalovirus promotor-controlled expression cassettes for the co-expression of two proteins. The expression constructs were further modified to contain coding sequences for the N-terminal signal peptides (e.g., MERHWIFLLLLSVTAGVHS) to facilitate secretion, which was also required for the type II membrane proteins. For recombinant fusion constructs (e.g., antigens with IgG1 Fc portion), sequences encoding fusion partners were PCR amplified using elongated primers with corresponding linker or connector sequences for gene fusion or restriction enzyme digestion. The resulting overlapping DNA fragments were inserted into the co-expression vector at the relevant position using Gibson Assembly^®^ Master Mix (New England Biolabs, Cat.no. E2611, Ipswich, MA, USA) to yield the final construct. Sequences of all constructs were confirmed by Sanger sequencing performed at Eurofins (Köln, Germany) using the custom-made primers. 

Site-directed biotinylation of recombinant antigens fused to Avi-Tag was performed using BirA biotin ligase (Biotin-Protein Ligase Kit, GeneCopoeia, Cat.no. BI001, Rockville, MD, USA), according to the manufacturer’s instruction. Reactions were performed in a BioRad Thermal Cycler T100 (BioRad, Dreieich, Hessen, Germany) for 1 h at 30 °C followed by buffer exchange using a Slide-A-Lyzer mini dialysis unit against 10 mM sodium-phosphate buffer, pH 7.4 (without potassium+) at 4 °C. Dialysis was performed three times for 1.5 h, 2.5 h, and overnight at 4 °C. For the quantitation of biotinylated proteins, the Pierce Biotin Quantitation Kit (Thermo Fisher Scientific, Cat.no. 28005, Waltham, MA, USA) was used, following the instructions in the manufacturer’s manual.

### 2.2. Biophysical Characterization 

Temperature-induced unfolding of purified recombinant proteins was measured by DSF on a Rotor-Gene Q (Qiagen, Hilden, Germany) applying a temperature gradient of 1 °C/minute. Melting temperatures, which were obtained by recording temperature-induced changes in Sypro^Ò^ Orange (Life Technologies, Cat. no. S6651, Eugene, OR, USA) fluorescence at 610 nm, were defined as a midpoint of the first observed protein unfolding transition.

To assess protein stability at 2–8 °C, 25 °C, or 40 °C for up to 7 days, at pH 3.5 for 90 min, and upon application of three freeze/thaw cycles (−80 °C/ambient temperature), samples were adjusted to 1.5 mg/mL concentration in 10 mM sodium acetate buffer supplied with 4.5% sorbitol (pH 5.0), incubated at each condition and analyzed by sodium dodecyl sulphate-polyacrylamide gel electrophoresis and SE-HPLC.

### 2.3. Surface Plasmon Resonance

Interaction kinetics of ICE^®^ construct binding to human CD16, EpCAM, NKp46, or NKG2D were analyzed at 37 °C using a Biacore T200 instrument (GE Healthcare, Uppsala, Sweden) equipped with a research-grade Sensor Chip CAP (Biotin CAPture Kit, Cytiva Sweden AB, Cat. no. 28920234, Uppsala, Sweden) pre-equilibrated in HBS-P+ running buffer. For monovalent interaction analysis, ICE^®^ constructs were captured on immobilized biotinylated recombinant monomeric human antigens (mFc.silenced/Avi-tagged CD16A^158V^, EpCAM, NKp46, or NKG2D) to a density of 20–60 RU before the corresponding recombinant monomeric human counter antigen (mFc.silenced/Avi-tagged CD16A^158V^ or CD16A^158F^, EpCAM, NKp46, or NKG2D) was injected at a concentration range of 0 to 240 nM for 180 s at a flow rate of 40 μL/minute and complex was left to dissociate for 300 s. For bivalent interaction analysis, biotinylated recombinant monomeric human antigens (mFc.silenced/Avi-tagged CD16A^158V^, CD16A^158F^, EpCAM, NKp46, or NKG2D) were captured to a density of 150 RU before ICE^®^ constructs were injected at a concentration range of 0 to 60 nM for 240 s at a flow rate of 40 μL/minute and complex was left to dissociate for 300 s. 

After each cycle, chip surfaces were regenerated with 6 M guanidine-HCl, 0.25 M NaOH and reloaded with Biotin Capture reagent. Interaction kinetics were determined by fitting data from multi-cycle kinetics experiments to a simple 1:1 interaction model using the local data analysis option (R_max_ and RI) available within Biacore T200 Evaluation Software v3.1 (Biacore, GE Healthcare, Uppsala, Sweden). Referencing was performed against a flow cell without a captured ligand (FC2-FC1, FC3-FC1, FC4-FC1).

### 2.4. ELISA

Antibody binding to recombinant antigen was analyzed using ELISA as previously described [24]. All antigens were fusion proteins of the extracellular domains and monomeric human Fc and were used for coating at a concentration range of 2.0–2.5 µg/mL (around 45–48 nM). Bispecific ICE^®^ constructs were titrated in a concentration range of 8 pM to 50 nM. To analyze weak interactions of the Fc portions of IgGs with different Fcg receptor variants, concentration ranges were expanded to 40 pM–1 µM. Bound antibodies were detected with anti-lambda light chain-horseradish peroxidase (Abcam, Cat. No. ab99811, Cambridge, UK). Absorbance values of 3,3′,5,5′-tetramethylbenzidine substrate reactions were measured at 450 nm using a multiwell plate reader (Ensight, Perkin Elmer, Waltham, MA, USA), plotted and analyzed using GraphPad Prism software version 9 (GraphPad Software, La Jolla, CA, USA). EC_50_ values were determined by fitting a nonlinear regression model (four parameters logistic fit) to sigmoidal dose–response curves. 

### 2.5. Cell Lines and Cell Culture

HCC-1187 (Cat. no. CRL-2322), HCC-1954 (Cat. no. CRL-2338), and Detroit 562 (Cat. no. CCL-138) cell lines were purchased from ATCC (Manassas, VA, USA). The KARPAS-299 cell line (Cat. no. ACC 31) was purchased from DSMZ (Braunschweig, Germany). Cells were cultured under standard conditions in the medium recommended by the vendor. Flp-In-CHO cells (Cat. no. R75807, Thermo Fisher Scientific, Waltham, MA, USA) were used for stable transfection as previously described [24].

### 2.6. Isolation of Peripheral Blood Mononuclear Cells and Enrichment of Human NK Cells

Peripheral blood mononuclear cells (PBMC) were isolated from the buffy coats (German Red Cross, Mannheim, Germany) of blood from healthy volunteers, as previously described [33,34]. NK cells were isolated from PBMC (STEMCELL Technologies, Inc., Grenoble, France, EasySEP™ Negative NK Cell Enrichment Kit, catalog number 17955), according to manufacturer’s instructions, and phenotyped by flow cytometry using CD16 158V-specific mAb MEM-154 (Fisher Scientific, catalog number MA1–19563).

### 2.7. Flow Cytometric Analysis of ICE^®^ Construct and Antibody Binding to Cells 

Aliquots of 0.2–1 × 10^6^ of the indicated cells were incubated with 100 μL of serial dilutions of the indicated ICE^®^ constructs and control antibodies in fluorescence-activated cell sorting (FACS) buffer (phosphate-buffered saline, Thermo Fisher Scientific, Cat. no. 14190–169, Waltham, MA, USA) containing 2% heat-inactivated fetal calf serum (FCS) (Thermo Fisher Scientific, Cat. no. 10270–106, Waltham, MA, USA) and 0.1% sodium azide (Roth, Cat. no. A1430.0100, Karlsruhe, Germany) for 45 min at 37 °C. After repeated washing with FACS buffer, cell surface-bound ICE^®^ constructs and antibodies were detected with 15 μg/mL fluorescein isothiocyanate (FITC)-conjugated goat anti-human IgG Fc (Dianova, Cat. no. 109-095-098, Hamburg, Germany), and fixable viability dye eF780 (Thermo Fisher Scientific, Cat. no. 65-0865-14, Waltham, MA, USA) was added to allow exclusion of dead cells. All incubations with secondary reagents and washing steps were performed on ice. After the last staining step, cells were washed again and resuspended in 0.2 mL of FACS buffer. The fluorescence of >1 × 10^4^ viable cells was measured using CytoFlex or CytoFlexS flow cytometers (Beckman Coulter, Krefeld, Germany), and the median fluorescence intensities (MFI) of cell samples were determined. After subtracting the fluorescence intensity values corresponding to cells stained with the secondary reagent alone, values were used for nonlinear regression analysis. Equilibrium dissociation constants (K_D_) were calculated using the one-site-binding (hyperbolic) fit and GraphPad Prism software version 9 (GraphPad Software, La Jolla, CA, USA).

### 2.8. Quantification of EpCAM Cell-Surface Expression

For the quantification of EpCAM expression on the surface of tumor cell lines, the specific antibody binding capacity (SABC) was determined after staining aliquots of the indicated cell lines with anti-EpCAM mAb 323/A3 (STEMCELL Technologies, Cat. no. 60146, Grenoble, France) at 10 µg/mL and anti-c-myc mAb 9E10 (Acris, Cat. no. SM1863P, Herford, Germany) at 10 µg/mL as a negative control using QIFIKIT (Agilent Technologies, Cat. no. K007811-8, Waldbronn, Germany), according to the manufacturer’s instructions, and subsequent flow cytometric analysis. 

### 2.9. Flow Cytometric Analysis of ICE^®^ Construct and Antibody Retention on the Surface of NK Cells 

Primary human NK cells were isolated and enriched by a negative selection as described [28]. Aliquots of cells were loaded with 100 µg/mL of the indicated ICE^®^ constructs and antibodies for 40–50 min on ice in RPMI 1640 medium (Cat. no. 21875–034) supplemented with 10% heat-inactivated FCS, 100 U/mL penicillin G/100 µg/mL streptomycin (Cat. no. 1540–122), and 2 mM L-glutamine (Cat. no. 25030–024; all from Thermo Fisher Scientific, Waltham, MA, USA), referred to as complete RPMI 1640 medium. After washing cells twice with chilled complete RPMI 1640 medium to remove unbound ICE^®^ constructs and antibodies, cell aliquots were resuspended in 10 mL of pre-warmed complete RPMI 1640 medium and incubated for 0, 0.5, 1, 2, 6, 24, and 48 h at 37 °C. Aliquots for timepoint 0 samples were immediately put on ice. For detection of retained ICE^®^ constructs and control antibodies, samples were washed twice with chilled FACS buffer and stained with FITC-conjugated goat anti-human IgG Fc (Dianova, Cat. no. 109-095-098, Hamburg, Germany), and fixable viability dye eF780 (Thermo Fisher Scientific, Cat. no. 65-0865-14, Waltham, MA, USA) was added for 40–50 min on ice. After washing cells twice with chilled FACS buffer, the MFI of >1 × 10^4^ viable cells was determined using CytoFlex or CytoFlexS flow cytometers (Beckman Coulter, Krefeld, Germany). For normalization, MFI determined for each antibody at timepoint 0 was set to 100%, MFI from NK cells stained with secondary reagent alone was set to 0%, and the relative amount of retained ICE^®^ constructs and antibodies was calculated. MFI from some samples were lower than MFI from stainings with secondary reagent alone, leading to negative values; values < −10% were excluded.

### 2.10. Cytotoxicity and NK-Cell Fratricide Assays

Calcein-release ADCC and NK cell fratricide assays were performed as previously described [24]. Briefly, for the calcein-release assay, target cells were labelled with calcein-AM (Invitrogen, cat.: C3100MP) for 30 min in RPMI media at 37 °C, washed, and seeded together in individual wells of a 96-well microplate with effector cells at the indicated effector:target (E:T) ratios. Effector and target cells were then incubated in the presence of increasing antibody concentrations. For NK cell fratricide assays, calcein-labelled primary human NK cells were incubated with autologous NK cells at an E:T ratio of 1:1 in the presence of increasing antibody concentrations. Following incubation at 37 °C in humidified conditions and 5% atmospheric CO_2_ for 4 h, calcein fluorescence in the supernatant was measured at 520 nm using an EnSight multimode plate-reader (Perkin Elmer, Turku, Finland). The specific cell lysis was then calculated. Mean values of specific target cell lysis (%) and standard devations (SD) were plotted and in vitro potency (EC_50_) was determined by fitting the nonlinear regression model to sigmoidal dose–response curves (variable slope) using GraphPad Prism (v7 and higher; GraphPad Software, La Jolla, CA, USA).

### 2.11. Pre-Complexing and Freeze/Thaw of NK Cell and ICE^®^ Complexes 

Primary human NK cells were isolated and enriched by a negative selection as previously described [28]. Aliquots of NK cells were pre-complexed with or without 10 µg/mL of the indicated ICE^®^ constructs or control antibodies in 0.5 mL of complete RPMI 1640 medium for 30 min at room temperature in 2 mL cryotubes. After adding 0.5 mL of FCS supplemented with 20% dimethylsulfoxide (Sigma-Aldrich, Cat. no. D2650-100mL, St. Louis, MO, USA) to each tube, cell suspensions were frozen at −80 °C for more than 12 h. For the assessment of the cytotoxic activity of pre-complexed NK cells, HCC-1187 and KARPAS-299 cells were used as target cells in 4 h calcein-release cytotoxicity assays as described [21]. Cryopreserved NK cells were thawed from −80 °C by incubation of cryotubes in a water bath at 37 °C. When the last ICE^®^ crystals were still visible, cell suspension was diluted with complete RPMI 1640 medium, centrifuged, and washed once before NK cell complexes were added to the target cells at the indicated effector-to-target ratio with or without 10 µg/mL of fresh antibody.

## 3. Results

### 3.1. Generation of Bispecific ICE^®^ Constructs and Their Biophysical Characterization

The ICE^®^ construct design was based on the tetravalent bispecific single-chain fragment variable (scFv) fusion antibodies specific for the human CD16A NK cell receptor and either the human EpCAM or CD30 antigen (Table S1). The variable heavy (VH) and variable light (VL) chain domains with the specificity for either the EpCAM (target domain 42) [35] or CD30 (target domain HRS3) antigen [34] were used in an effector function silent human IgG1 scaffold. The VH and VL chain domains from the anti-CD16A effector domain P2C-47 [24,36] were fused to the C-terminus of the CH3 domain in the scFv (VL–VH domain orientation) format using a peptide linker. To generate control antibodies, the VH and VL domains from the EpCAM-specific target domain 42 were fused at their C-termini to the CH1 and CL domains of effector-enhanced human IgG1 [37] or wild-type human IgG1 (Table S1). ICE^®^ constructs and control antibodies were expressed in Chinese hamster ovary (CHO) cells and purified from clarified cell culture supernatants [24]. The purity of preparations used for further characterization was determined by using the analytical size-exclusion high-performance liquid chromatography (SE-HPLC) and ranged from 91.5% to 98.2%, with no more than 2.4% of high molecular weight forms detected in these preparations (data not shown). 

Differential scanning fluorometry (DSF) evaluated the thermal stability of bispecific ICE^®^ constructs and identified temperatures of 54 °C and above as the first transition midpoint of protein denaturation, thus confirming the thermal stability of all ICE^®^ constructs (Table S2 and Figure S1). When tested for stability at different conditions, ICE^®^ constructs showed no substantial changes in a relative product content (>94%) after the incubations at 4–8 °C, 25 °C, and 40 °C temperatures for 7 days, or at pH 3.5 for 90 min, and after being subjected to three freeze and thaw cycles (Table S3) [24]. 

### 3.2. Surface Plasmon Resonance-Measured Kinetics of the Binding of ICE^®^ Constructs to EpCAM and NK Cell Receptors 

**Figure 1:**
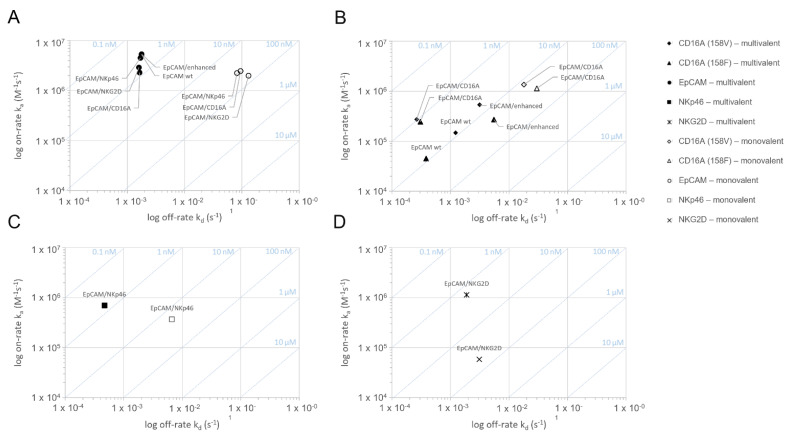
Assessment of the monovalent and multivalent binding kinetics of bispecific ICE^®^ constructs to recombinant human EpCAM, CD16A, NKG2D, and NKp46 antigens: on-/off-rate map.The binding interaction of EpCAM/CD16A, EpCAM/NKp46, EpCAM/NKG2D, anti-EpCAM IgG1, and anti-EpCAM Fc-enhanced IgG1 was measured on human EpCAM (**A**), human CD16A^158V/F^ (**B**), human NKp46 (**C**), and human NKG2D (**D**) in SPR at 37 °C using an experimental set-up allowing for the monovalent or multivalent interaction (*n* = 3). Binding curves were fitted to a 1:1 binding model to evaluate the kinetic rate constants k_a_ and k_d_ as well as a dissociation equilibrium constant K_D_. The arithmetic means were calculated and plotted. SPR stands for surface plasmon resonance.

The ICE^®^ constructs specific for EpCAM and CD16A^158V^, CD16A^158F^, NKp46, or NKG2D were tested using surface plasmon resonance (SPR) for retention and strength of their binding to the respective target antigens (Figure 1 and Table 1). To measure the monovalent interaction kinetics, bispecific ICE^®^ constructs were captured on immobilized monomeric biotinylated recombinant EpCAM, CD16A, NKp46, or NKG2D proteins and the binding affinity was analyzed with the respective recombinant human proteins. Simultaneous and specific monovalent binding to both antigens occurred (Figure 1 and Table 1). The monovalent binding affinity (K_D_) ranking for interactions between specific effector domains and respective ICE^®^ constructs was CD16A^158V^ > NKp46 > CD16A^158F^ > NKG2D, with the highest affinity (K_D_ 13.9 nM) identified for CD16A^158V^ and the lowest (K_D_ 53.6 nM) for NKG2D (Figure 1 and Table 1).

Multivalent interaction analyses, performed using SPR, also confirmed the multivalent binding of bispecific ICE^®^ constructs to their target antigens (Figure 1 and Table 1), and the functionality of these interactions was proven by observing an increase in the apparent affinity (K_D_) compared with the monovalent apparent binding affinity by 55–114-fold for EpCAM, 14–23-fold for CD16A, 26-fold for NKp46, and 32-fold for NKG2D (Table 1). Therefore, SPR analyses confirmed that all ICE^®^ constructs bind bivalently to their respective target antigens (Table 1). 

EpCAM-specific ICE^®^ constructs and IgG1 control antibodies showed very high and comparable apparent binding affinities to human EpCAM (multivalent K_D_ 0.3–0.7 nM) (Table 1), thus proving the functionality of the anti-EpCAM fragment variable (Fv) in the fragment antigen-binding (Fab) region arranged in the identical orientation in these molecules. Furthermore, the EpCAM/CD16A ICE^®^ construct showed a similar affinity to both CD16A^158V^ and CD16A^158F^ alleles (1.0 vs. 1.2 nM) (Table 1), which is consistent with the results of a study that reported the binding kinetics of different antibodies derived from the ROCK^®^ platform [24]. The affinity of the EpCAM/CD16A ICE^®^ construct to CD16A^158V^ and CD16A^158F^ alleles was higher than that of anti-EpCAM IgG1 and anti-EpCAM Fc-enhanced IgG1 controls (Table 1). These data prove that ICE^®^ constructs bind selectively and bispecifically to their respective target antigens through both monovalent and multivalent interactions.

### 3.3. Assessment of the Binding and Specificity of ICE^®^ Constructs to NK Cell Receptors Measured by Enzyme-Linked Immunosorbent Assay

**Figure 2:**
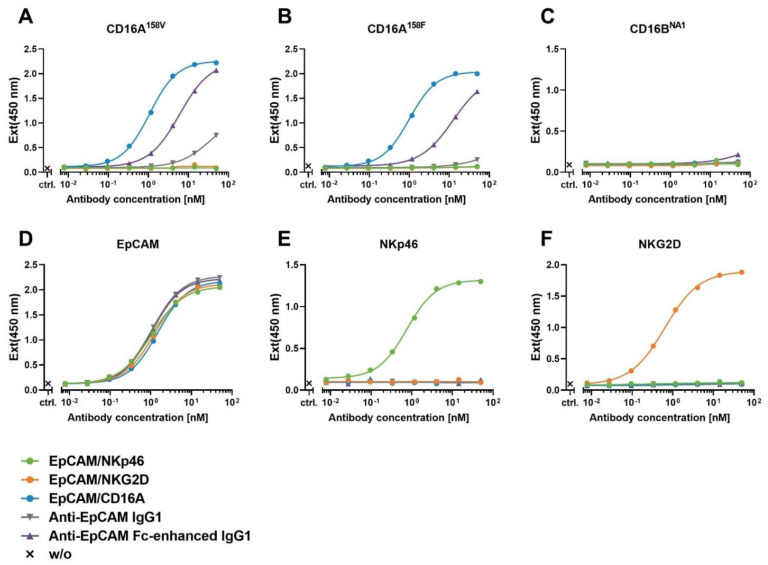
Assessment of the binding and specificity of bispecific ICE^®^ constructs to recombinant human CD16A, CD16B, EpCAM, NKp46, or NKG2D antigens measured by ELISA.ELISA was used to analyze the binding of ICE^®^ constructs or control IgG antibodies to plates coated with human CD16A^158V^ (**A**), CD16A^158F^ (**B**), CD16B^NA1^ (**C**), EpCAM (**D**), NKp46 (**E**), or NKG2D (**F**). Bound antibodies were detected with the HRP-conjugated anti-lambda light chain-secondary antibody and measured in TMB substrate reactions at 450 nm. ELISA, enzyme-linked immunosorbent assay; Ext, excitation; HRP, horseradish peroxidase; TMB, 3,3′,5,5′-tetramethylbenzidine.

To confirm the specificity of binding and determine the binding affinity, ICE^®^ constructs were tested in the enzyme-linked immunosorbent assay (ELISA) using immobilized fusion proteins comprising the extracellular domains of CD16A^158V^, CD16A^158F^, CD16B, EpCAM, NKp46, or NKG2D and monomeric human Fc. EpCAM-specific ICE^®^ constructs showed antigen-specific high-affinity binding to EpCAM and respective NK-cell receptor epitopes, and no cross-reactivity with other receptors used in this assay was detected (Figure 2 and Table 2). The EpCAM/CD16A ICE^®^ construct bound both human CD16A allotypes (158V and 158F) with a similar affinity, which is consistent with the reported binding characteristics of other CD16A-specific ICE^®^ constructs [24], whilst anti-EpCAM IgG1 and Fc-enhanced control antibodies showed a lower affinity to these CD16A variants (Figure 2 and Table 2). The EpCAM/CD16A ICE^®^ bound with a lower affinity to CD16A than EpCAM/NKp46 and EpCAM/NKG2D ICE^®^ constructs to NKp46 and NKG2D, respectively (Table 2). The apparent binding affinities of EpCAM-specific ICE^®^ constructs and control antibodies to immobilized EpCAM were comparable (Table 2). These results confirm the specificity of ICE^®^ construct binding to respective target antigens seen also in the SPR experiments.

### 3.4. Binding of ICE^®^ Constructs to NK Cell Receptors Expressed on the Surface of CHO Cells and to Primary Human NK Cells

To examine whether the specificity and high-affinity binding of ICE^®^ constructs for NK cell receptors is maintained when tested on CHO cells engineered to express these receptors, ICE^®^ constructs were titrated for binding to CHO cells expressing recombinant human CD16A^158F^, CD16A^158V^, CD16B^NA1^, NKp46, or NKG2D. All ICE^®^ constructs showed specific and high-affinity binding to CHO cells expressing respective NK cell receptors (Table 3). Consistently with the ELISA results, the EpCAM/NKp46 and EpCAM/NKG2D ICE^®^ bound recombinant NKp46 and NKG2D on the surface of CHO cells with a higher affinity (K_D_ 0.6 and 1.7 nM, respectively) than the EpCAM/CD16A ICE^®^ to CD16A alleles on CHO cells (K_D_ 27.7 and 22.7 nM) (Table 3), with the EpCAM/NKp46 ICE^®^ showing a 13-fold higher affinity and the EpCAM/NKG2D ICE^®^ a nearly 38-fold higher affinity (Table 3). EpCAM/CD16A and CD30/CD16A ICE^®^ constructs exhibited similar apparent affinities to CD16A^158F^- and CD16A^158V^-expressing CHO cells, with the mean K_D_ values of 27.7 nM and 24.9 nM for the CD16A^158F^ variant and 22.7 nM and 22.5 nM for the CD16A^158V^ variant, respectively (Table 3). The anti-EpCAM IgG1 control antibody exhibited substantially lower affinities to both CD16A alleles than the EpCAM/CD16A ICE^®^ construct (Table 3), whilst the Fc-enhanced antibody had higher affinities than anti-EpCAM IgG1 for CD16A^158V^-expressing (26.3 nM vs. 308 nM) and CD16A^158F^-expressing CHO cells (80.7 nM vs. 889 nM) (Table 3). Specifically, the EpCAM/CD16A ICE^®^ exhibited a 32-fold higher affinity for CD16A^158F^ than anti-EpCAM IgG1 and an approximately 3-fold higher affinity than Fc-enhanced IgG1, and a 14-fold higher affinity for the CD16A^158V^ variant than anti-EpCAM IgG1, and similar affinity as Fc-enhanced IgG1 (Table 3). In contrast to the anti-EpCAM IgG1 and Fc-enhanced IgG1 control antibodies, the EpCAM/CD16A ICE^®^ showed no binding to the CD16B^NA1^ receptor expressed on the surface of CHO cells (Table 3).

**Figure 3:**
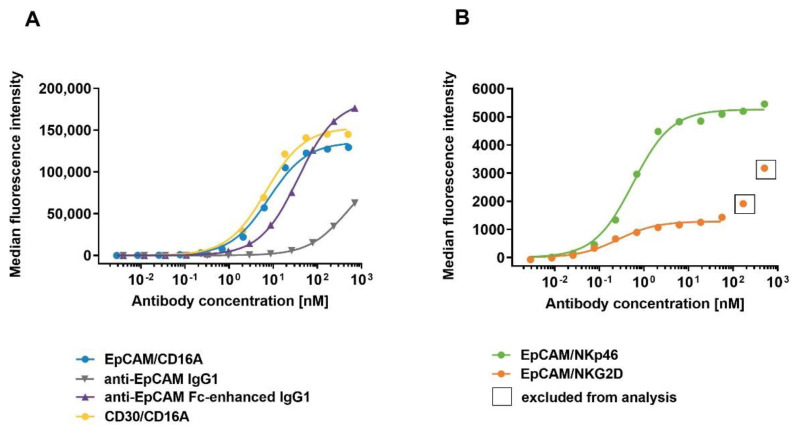
Binding of ICE^®^ constructs and control antibodies to primary human NK cells. Enriched primary human NK cells were incubated with serial dilutions of anti-EpCAM/CD16A ICE^®^, anti-EpCAM IgG1, anti-EpCAM Fc-enhanced IgG1, and CD30/CD16A ICE^®^ as a control (**A**), and EpCAM/NKp46 and EpCAM/NKG2D ICE^®^ constructs (**B**) at 37 °C. The cell-surface bound ICE^®^ constructs and control antibodies were detected with FITC-conjugated goat anti-human IgG Fc and the flow cytometric analysis. Data from a single representative experiment out of three experiments are presented. FITC, fluorescein isothiocyanate.

To investigate the binding affinities of ICE^®^ constructs to NK cells, EpCAM- and CD30-specific ICE^®^ constructs and control anti-EpCAM antibodies were titrated on primary human NK cells and cell surface-bound antibody constructs were quantified by flow cytometry. All ICE^®^ constructs and control anti-EpCAM IgG1 antibodies showed concentration-dependent binding to human NK cells at 37 °C (Figure 3). EpCAM/NKG2D and EpCAM/NKp46 ICE^®^ constructs exhibited the highest apparent affinity (mean K_D_ 0.2 nM and 0.5 nM, respectively) (Table 4), although the MFI were the lowest of all tested molecules at all concentration levels (Figure 3). This suggests that the NK cell-surface expression levels of NKG2D and NKp46 are substantially lower than those of the CD16A receptor. MFI levels for the NKp46-specific ICE^®^ were generally higher than those of the NKG2D-specific ICE^®^ possibly due to relatively higher NKp46 expression levels on the surface of NK cells (Figure 3B). The apparent affinity of the EpCAM/CD16A ICE^®^ was nearly 16-fold and 40-fold lower than that of the EpCAM/NKp46 ICE^®^ and EpCAM/NKG2D ICE^®^, respectively (Table 4). Both CD16A-specific ICE^®^ constructs (EpCAM/CD16A and CD30/CD16A) exhibited similar binding affinities to NK cells (Figure 3A), with the mean K_D_ values of 7.9 nM and 6.7 nM, respectively (Table 4). These results confirm the ability of ICE^®^ constructs to effectively bind NK cells, with the higher affinity for the NKp46 and NKG2D receptors and a relatively lower affinity for the CD16A receptor.

### 3.5. Binding of EpCAM-Specific ICE^®^ Constructs to EpCAM^+^ Tumor Cell Lines 

**Figure 4:**
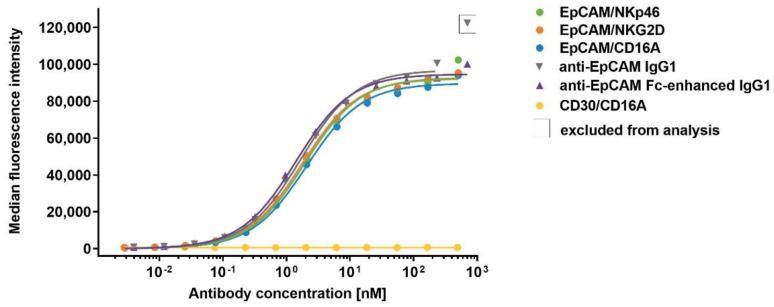
Concentration-dependent binding of anti-EpCAM ICE^®^ constructs and anti-EpCAM IgG1 antibodies to EpCAM^+^ tumor cells. EpCAM^+^ HCC-1187 tumor cells were incubated with serial dilutions of EpCAM/NKp46, EpCAM/NKG2D, EpCAM/CD16A, anti-EpCAM IgG1, anti-EpCAM Fc-enhanced IgG1, and CD30/CD16A ICE^®^ as a control at 37 °C. The cell-surface bound antibody constructs were detected with FITC-conjugated goat anti-human IgG Fc and flow cytometric analysis. Data for one representative experiment out of three experiments are presented. FITC, fluorescein isothiocyanate.

To confirm that EpCAM-specific ICE^®^ constructs and control antibodies can effectively target EpCAM-expressing tumor cell lines, EpCAM-specific ICE^®^ constructs and IgG1 control antibodies were titrated at increasing concentrations for binding to the HCC-1187, HCC-1954, and Detroit 562 tumor cell lines that express relatively high levels of EpCAM (Table S4), and cell surface-bound constructs were analyzed by flow cytometry. EpCAM-specific ICE^®^ constructs exhibited the concentration-dependent binding to EpCAM^+^ cell lines as shown for the HCC-1187 cells in Figure 4, whilst the CD30/CD16A ICE^®^ control showed no binding (Figure 4 and Table 5). EpCAM-specific ICE^®^ constructs bound with a similarly high apparent affinity to EpCAM^+^ tumor cell lines and with the K_D_ values ranging from 1.2 nM to 2.0 nM (Table 5). The mean specific antibody binding capacity (SABC) was 111,931 for the HCC-1187 cells, 184,253 for the HCC-1954 cells, and 215,240 for the Detroit 562 cells (Table S4). Despite the EpCAM expression level variability detected on the surface of these cancer cell lines, the apparent binding affinities of different EpCAM-specific ICE^®^ constructs were similar (Table 5). Since all EpCAM-specific ICE^®^ constructs have an identical anti-EpCAM Fv domain in their Fab arms and bind to EpCAM on tumor cells equally well, the biological activity of bridging tumor cells with the effector NK cells by these constructs is expected to be determined by the specificity and affinity of NK cell recruiting domains to NK cell receptors.

### 3.6. Assessment of NK Cell Fratricide Induced by the Binding of ICE^®^ Constructs and Anti-EpCAM IgG1 Antibodies to CD16A, NKp46, and NKG2D 

ICE^®^ constructs with different target specificity comprise two binding sites for each NKp46, NKG2D, or CD16A receptors, thereby potentially enabling a bivalent binding of ICE^®^ molecules to the receptors expressed on separate NK cells and consequently crosslinking these cells to cause NK cell fratricide. A previous study reported the anti-CD38 antibody, daratumumab, designed to target CD38-expressing myeloma cells, to induce NK cell depletion in patients with multiple myeloma and suggested that the mechanism by which daratumumab reduces the number of NK cells is NK cell fratricide [38] mediated by cross-linking of NK cells via cell-surface CD38. 

**Figure 5:**
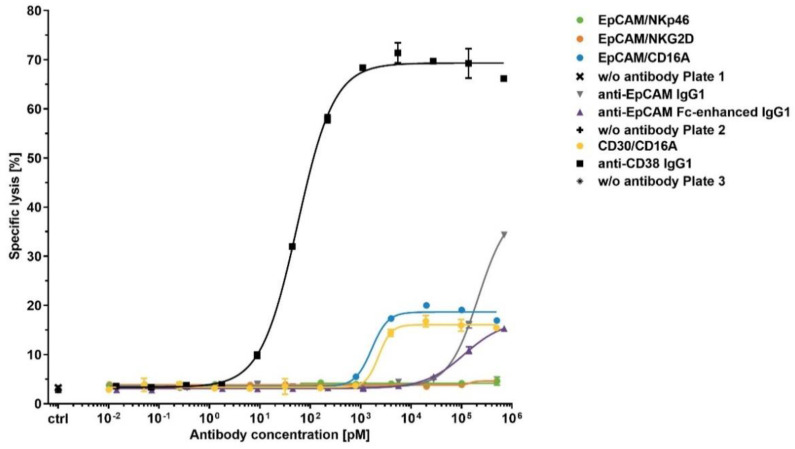
Assessment of NK cell fratricide induced by bispecific ICE^®^ constructs and anti-EpCAM IgG1. Calcein-labeled enriched primary human NK cells were co-cultured with autologous NK cells as effector cells at an E:T ratio of 1:1 in the presence of indicated antibodies at serial dilutions. After a 4 h incubation, the release of fluorescent calcein from lysed target cells into the supernatant was quantified and used for calculation of the percentage of specific lysis. Anti-CD38 IgG1 was used as a positive control. Mean of duplicate values ± standard deviation are plotted.

To test the potential of ICE^®^ constructs to induce NK cell fratricide, calcein-labeled NK cells served as target cells and the autologous NK cells as effector cells. Cells were mixed at an effector:target cell ratio of 1:1 and then tested in a 4 h calcein-release cytotoxicity assay. A human IgG1 containing daratumumab-derived anti-CD38 Fv domain was used as a positive control in all NK cell fratricide assays and showed the mean potency (EC_50_) value of 73.6 pM and the mean efficacy (E_max_) of 61.1% (Figure 5 and Table 6). 

All CD16A-specific ICE^®^ constructs induced measurable but moderate NK cell lysis, with low potencies in a nanomolar concentration range, whilst the CD30-specific ICE^®^ exhibited approximately 2-fold higher E_max_ (mean E_max_ 21.7%) than the EpCAM-specific ICE^®^ that had the mean E_max_ of only 11.8% (Table 6). Furthermore, the NKp46- and NKG2D-specific ICE^®^ constructs induced no NK cell fratricide under the same assay conditions, despite the higher apparent affinities for the respective NK receptors identified by flowcytometric analyses (Figure 3 and Table 4). The mean E_max_ of anti-EpCAM IgG1 and anti-EpCAM Fc-enhanced IgG1 controls were 13.1% and 9.0%, respectively (Table 6). These results suggest that, despite both CD16A-specific ICE^®^ constructs being able to induce low levels of NK cell fratricide, the potencies and efficacies to mediate NK cell lysis are within the acceptable range to continue further preclinical development of such ICE^®^ constructs.

### 3.7. Target Tumor Cell Lysis Induced by ICE^®^ Constructs 

**Figure 6:**
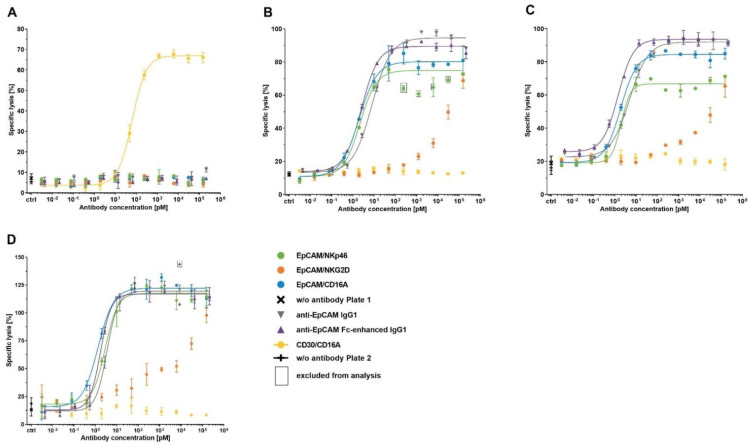
Specificity of bispecific ICE^®^ constructs to induce EpCAM^+^ tumor cell lysis. Calcein-labeled KARPAS-299 (**A**), HCC-1954 (**B**), Detroit 562 (**C**), and HCC-1187 cells (**D**) were co-cultured with enriched primary human NK cells as effector cells at an E:T ratio of 5:1 in the presence of serial dilutions of the indicated antibodies. After a 4 h incubation, the levels of calcein fluorescence released from the lysed target cells into the supernatant were quantified and used for the calculation of the percentage of specific lysis. Mean of duplicate values ± standard deviation are plotted. For specific constructs in different cell lines, fitting of sigmoidal dose–response curves was not possible. E:T, effector-cell-to-target-cell ratio.

To investigate the potency and efficacy of ICE^®^ constructs to mediate destruction of target tumor cells, EpCAM-specific ICE^®^ constructs, anti-EpCAM IgG1 control antibodies and the CD30-specific ICE^®^ were subjected to standard 4 h calcein-release cytotoxicity assays using EpCAM^+^/CD30^−^ tumor cell lines (HCC-1954, Detroit 562, and HCC-1187) and the EpCAM^−^/CD30^+^ KARPAS-299 cell line. All ICE^®^ constructs and IgG1 antibody controls mediated NK cell induced specific lysis of tumor cells expressing respective target antigens in a concentration-dependent manner (Figure 6). Tumor cell lysis was strictly dependent on the expression of a specific target antigen (Figure 6), demonstrating that the bivalent engagement of NK cells does not lead to off-target NK cell activation and lysis of antigen-negative cells. 

Although EpCAM/NKG2D ICE^®^-mediated EpCAM^+^ target tumor cell lysis showed a concentration-dependent pattern, the calculation of mean EC_50_ and E_max_ values was not feasible due to a nonlinear regression model producing no sigmoidal dose–response curves (Figure 6).

ICE^®^ constructs targeting the CD16A NK cell receptor induced a more extensive EpCAM^+^ tumor cell destruction than the EpCAM/NKp46 ICE^®^ (Table 7), despite a lower binding affinity to CD16A (Figure 3 and Table 4). Across all EpCAM^+^ tumor cell lines, the EpCAM/CD16A ICE^®^ exhibited the highest potency, and the EpCAM/NKp46 ICE^®^ and anti-EpCAM Fc-enhanced IgG1 had potencies falling in the similar concentration range (Table 7). However, although anti-EpCAM IgG1 was highly effective in inducing EpCAM-specific tumor cell lysis, its potency was substantially lower compared with that of the EpCAM/CD16A and EpCAM/NKp46 ICE^®^ constructs and anti-EpCAM Fc-enhanced IgG1 (Table 7). Furthermore, there seemed to be a correlation between the potency of anti-EpCAM IgG1 and EpCAM expression levels on the cell surface of tumor cells. The highest potency (mean EC_50_ 6.6 pM) was determined for the Detroit 562 target cells expressing the highest levels of EpCAM, and the lowest potency (mean EC_50_ 40.1 pM) for the HCC-1187 cells expressing the lowest levels of EpCAM (Table 7 and A4). These results demonstrate the efficacy of ICE^®^ constructs to mediate tumor cell lysis with high potency and in a strictly antigen-dependent manner.

### 3.8. Assessment of the Cell-Surface Retention of ICE^®^ Constructs on Primary NK Cells

**Figure 7:**
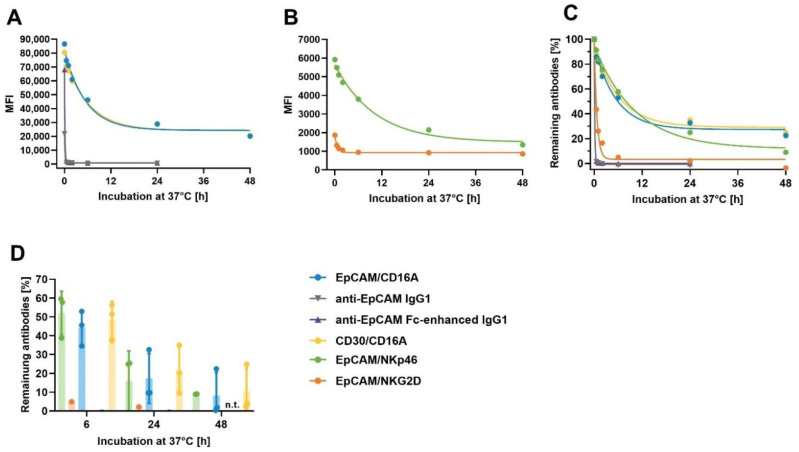
Retention of anti-EpCAM ICE^®^ and IgG1 antibodies on NK cells at 37 °C. Aliquots of enriched primary human NK cells were pre-complexed with 100 µg/mL of EpCAM/NKp46, EpCAM/NKG2D, EpCAM/CD16A, anti-EpCAM IgG1, anti-EpCAM Fc-enhanced IgG1, and CD30/CD16A as a control on ICE^®^. After removal of unbound antibodies, aliquots were either directly stained with FITC-conjugated goat anti-human IgG1 Fc on ICE^®^ and analyzed by flow cytometry, or incubated for 0.5, 1, 2, 6, 24, and 48 h at 37 °C before detection of retained antibodies by flow cytometry. After subtracting the MFI of NK cells incubated with the secondary reagent alone at the time point 0, MFI for retained EpCAM/CD16A ICE^®^, anti-EpCAM IgG1, anti-EpCAM Fc-enhanced IgG1, and CD30/CD16A ICE^®^ (**A**), and EpCAM/NKp46 and EpCAM/NKG2D ICE^®^ constructs (**B**) were plotted. The percentage of retained ICE^®^ constructs and IgG1 antibodies relative to initially bound ICE^®^ constructs and IgG1 antibodies were plotted (**C**). One out of three representative experiments is presented (**A**–**C**). The relative amount of retained antibodies after 6, 24, and 48 h incubations at 37 °C determined in three independent experiments is shown in (**D**). The MFI for some samples were lower than MFI for staining with the secondary reagent alone, leading to negative values; values < −10% were excluded. Where applicable, the individual and mean values ± standard deviation derived from three independent experiments are shown. FITC, fluorescein isothiocyanate; MFI, median fluorescence intensity; n.t., not tested.

A strong and stable interaction between the ICE^®^ constructs and effector cells is a prerequisite for optimal cryopreservation of such complexes, without the loss of activity. The strong binding affinity (K_D_ 0.2–7.9 nM) to primary NK cells was established for all effector binding domains, as described in Section 2.4 (Table 4). In order to confirm the stability and slow off-rates for the breakdown of ICE^®^ and NK cell complexes, the degree of retention of ICE^®^ constructs and control antibodies on the surface of NK cells after pre-complexing was assessed. ICE^®^ constructs and anti-EpCAM IgG1 control antibodies were allowed to dissociate from NK cells at 37 °C for different time periods, and the levels of remaining antibodies bound to NK cells were analyzed by flow cytometry. A very fast dissociation of anti-EpCAM IgG1 and anti-EpCAM Fc-enhanced IgG1 controls occurred, with the relative amount of retained antibodies being below 1% after a 1 h dissociation period (Figure 7A). Despite a very high apparent affinity of the EpCAM/NKG2D ICE^®^ to the cell-surface NKG2D receptor (Figure 3 and Table 4), it also displayed fast dissociation, and the amount of retained EpCAM/NKG2D ICE^®^ was below 5% after 6 h from pre-complexing (Figure 7B). Interestingly, despite the lower apparent affinity of EpCAM/CD16A and CD30/CD16A ICE^®^ constructs for CD16A compared with those of the EpCAM/NKG2D ICE^®^ to NKG2D (Figure 3 and Table 4), both CD16A- and NKp46-specific ICE^®^ constructs showed similar dissociation kinetics (Figure 7C). A relatively high assay-to-assay variability was observed after the longer dissociation phases, partially due to low cell-surface expression levels of these receptors (Figure 7D). The mean values for the proportion of EpCAM/NKp46, EpCAM/CD16A and CD30/CD16A ICE^®^ constructs remaining bound to NK cell surface after 24 h ranged between 15.9% and 21.6% and after 48 h, between 8.2% and 10.4% (Table S5). These data suggest that CD16A- and NKp46-specific ICE^®^ constructs might be suitable for pre-complexing with NK cells and cryopreservation.

### 3.9. Cytotoxic Activity of ICE^®^-Pre-Complexed NK Cells after Reconstitution from Cryopreservation 

**Figure 8:**
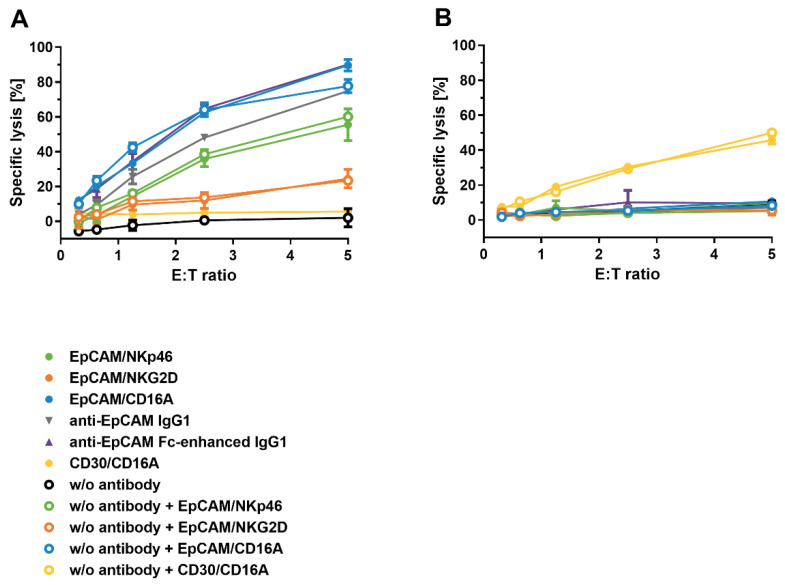
Cytotoxicity of cryopreserved NK cells pre-complexed with ICE^®^ constructs against HCC-1187 and KARPAS-299 target cells. NK cells as effector cells were pre-complexed with 10 µg/mL of the indicated ICE^®®^ constructs and antibodies and frozen at −80 °C. As control samples, aliquots of the same NK cells were pre-incubated without an antibody (w/o antibody) and subjected to one freeze/thaw cycle. Thawed NK cells were washed and directly used as effector cells at the indicated E:T ratios. Where indicated, ICE^®^ constructs (EpCAM/NKp46, EpCAM/NKG2D, EpCAM/CD16A, or CD30/CD16A) were freshly added to NK cells that were not pre-complexed at a concentration of 10 µg/mL to the cytotoxicity assays against HCC-1187 (**A**) and KARPAS-299 (**B**) target cells. The mean ± standard deviation of duplicate lysis values were plotted. E:T, effector-cell-to-target-cell ratio; w/o, without.

To assess how cryopreservation affects the ability of ICE^®^ constructs pre-complexed with NK cells to induce NK cell mediated tumor cell destruction, primary human NK cells pre-complexed with ICE^®^ constructs and control antibodies were revived to test their activity against specific tumor cells using calcein-release cytotoxicity assays. As shown in Figure 8, NK cells pre-complexed with EpCAM-specific ICE^®^ constructs or anti-EpCAM control antibodies were effective in inducing lysis of the EpCAM^+^ HCC-1187 cells, whilst they were inactive against the EpCAM^−^/CD30^+^ KARPAS-299 cells. In contrast, revived NK cell and CD30/CD16A ICE^®^ complexes induced the effective lysis of the EpCAM^−^/CD30^+^ KARPAS-299 cells, but not of EpCAM^+^/CD30^−^ HCC-1187 cells (Figure 8). Among the EpCAM-specific ICE^®^ constructs, NK cells pre-complexed with the EpCAM/CD16A ICE^®^ displayed the highest efficacy in lysing the HCC-1187 cells (74.8%), with the EpCAM/NKp46 ICE^®^ medium efficacy (46.1%), and with the EpCAM/NKG2D ICE^®^ the lowest efficacy (14.3%) (Table 8). Interestingly, revived NK cell and anti-EpCAM IgG1 or anti-EpCAM Fc-enhanced IgG1 complexes were also effective in lysing EpCAM^+^ target cells, and the efficacy of these complexes fell within a similar range as that of the EpCAM/CD16A ICE^®^, despite the low apparent binding affinity to the CD16A receptor (Figure 3 and Table 4) and fast dissociation from NK cells (Figure 7). NK cells pre-complexed with ICE^®^ constructs, when frozen, thawed, and washed, induced similar degrees of target cell lysis as NK cells without pre-complexing, but supplemented with fresh ICE^®^ constructs in the cytotoxicity assay (Table 8). These data provide strong evidence that, upon cryopreservation, ICE^®^ constructs pre-complexed with NK cells retain their cytotoxicity and specificity to a similar degree as non-cryopreserved complexes.

## 4. Discussion

A robust and effective methodology for pre-complexing of bispecific ICE^®^ constructs with primary NK cells (CAR-like NK-cell products) and cryopreservation of such complexes for adoptive transfer without a loss of specificity and cytotoxicity could significantly advance the development of off-the-shelf NK cell products, thereby circumventing the drawbacks of personalized NK cell therapies for patients with cancer. Moreover, an allogeneic off-the-shelf NK cell approach could reduce the manufacturing time and costs, ensuring timely delivery of these therapies to a larger patient population [39].

This study provides proof of the concept that human adoptive NK cells pre-complexed with bispecific ICE^®^ constructs, especially those that target the CD16A and NKp46 receptors, are suitable for cryopreservation, and that revived complexes retain their specificity and cytotoxicity against tumor cells expressing specific target antigens.

The stability of therapeutic antibodies is key for the successful development of effective antibody-based therapies. The ICE^®^ constructs investigated in this study were thermally stable and showed robust behavior at different temperatures, acidic pH conditions and after multiples freeze and thaw cycles. Melting profiles of bispecific ICE^®^ assessed by DSF showed transition events at approximately 62 °C, which is suggested to incorporate unfolding of IgG1 CH2 domain as well as of the fused scFv, and at 72 °C representing unfolding of Fab regions, which are in accordance with the literature and expected for IgG-like molecules [40]. An additional early transition event at 54.0 °C for EpCAM/NKp46 ICE^®^ is suggested to be derived from anti-NKp46 scFv. The early unfolding of Fc engineered IgG1, including the mutation S239E/I332D, is also well known and has been published [41].

The different experimental setups explored in this study, such as ELISA and the ICE^®^ binding to CHO cells expressing specific antigens and to NK cells, consistently demonstrated a similar specificity and binding affinity pattern, with the EpCAM/NKG2D ICE^®^ showing the highest affinity, whilst the EpCAM/CD16A ICE^®^ showed the lowest. Both CD16A-specific ICE^®^ constructs (EpCAM/CD16A and CD30/CD16A) showed a 3–4-fold stronger binding to primary NK cells than to CHO cells expressing recombinant CD16A. In general, MFI levels of the NK cell-surface bound EpCAM/NKp46 and EpCAM/NKG2D ICE^®^ were lower than those of the EpCAM/CD16A ICE^®^ perhaps due to the differing abundance of these receptors on the surface of primary human NK cells, such as the mean SABC/cell of 70,000 for CD16A, 3000–4000 for NKp46, and 1000–2000 for NKG2D, as reported in a previous study [42].

ICE^®^ constructs bound NK cells independently of the CD16A receptor allotype (158 V/F), which is consistent with the results of an earlier study that investigated the binding properties of other ICE^®^ constructs derived from the ROCK^®^ platform [24]. For monoclonal therapeutic antibodies, several clinical studies found a correlation between the CD16 polymorphisms and antibody efficacy in inducing ADCC. The CD16^158V/V^ variant in patients with metastatic colorectal cancer was reported to be a prognostic factor for disease progression in response to treatment with the combination of cetuximab and chemotherapy [43]. Trastuzumab showed higher in vitro cytotoxicity against peripheral blood mononuclear cells carrying the CD16^158V/V^ than CD16^158V/F^ or CD16^158F/F^ variants, although only one clinical study in patients with metastatic human epidermal growth factor receptor 2-positive breast cancer treated with a combination of trastuzumab and a taxane showed a correlation between the CD16^158V/V^ phenotype and an improved objective response rate and prolonged progression-free survival [43,44]. Several small studies in patients with lymphoma demonstrated the CD16^158V/F^ polymorphisms to predict a response to single agent rituximab [43].

Despite having the lowest apparent binding affinity to NK cells, the EpCAM/CD16A ICE^®^ showed superior cytotoxicity compared with that of the EpCAM/NKG2D and EpCAM/NKp46 ICE^®^ constructs against EpCAM^+^ tumor cells potentially due to higher cell-surface expression levels of CD16A and/or stronger NK cell activation upon CD16A ligation. This was particularly evident after a single freeze–thaw cycle, suggesting that both the binding affinity of ICE^®^ to the NK cell and the type and signaling pathway of the activating NK cell receptors involved, might contribute to the anti-tumoral activity of ICE^®^ stimulated NK cells. Following cryopreservation, revived EpCAM/NKp46 ICE^®^ and NK cell complexes were more potent activators of specific tumor cell lysis than the EpCAM/NKG2D ICE^®^ complexes. Another study reported that in vitro, anti-NKp46 monoclonal antibodies were more potent activators of the NK cell effector function than anti-NKG2D monoclonal antibodies [21].

The multivalent nature of an interaction between bispecific ICE^®^ constructs and NK cells determines the slower dissociation kinetics and more durable complex formation, which allows more efficient reconstitution from cryopreservation, without any loss of the high ADCC potential. NK cell cytotoxicity is a complex function, which depends on a simultaneous engagement and activation of multiple NK cell receptors. The activation of LFA-1, NKG2D, and 2B4 receptors was shown to be a minimal requirement for natural cytotoxicity, whilst CD16 was suggested to be the only receptor that is sufficient to induce cytotoxicity by the resting NK cells [42,45].

NK cell fratricide might pose significant limitations on the therapeutic index of novel NK cell therapies and immunotherapies. Importantly, this study provided strong evidence that ICE^®^ constructs have minimal capacity to induce NK cell fratricide. Both EpCAM/NKp46 and EpCAM/NKG2D ICE^®^ constructs were unable to induce NK cell lysis, whilst CD16A-specific ICE^®^ constructs displayed low potency and efficacy in inducing NK cell fratricide. One plausible explanation for this activity might relate to significantly higher CD16A than NKp46 and NKG2D expression levels on the surface of NK cells [42], which may increase the likelihood of the NK cell crosslinking and NK cell fratricide in this in vitro assay setup. The CD30/CD16A ICE^®^ showed even higher efficacy than the EpCAM/CD16A ICE^®^ to mediate NK cell lysis and the underlying cause for this might be a low-level CD30 expression on NK cells associated with potentially marginal NK cell activation triggered by the CD16A-specific ICE^®^.

Trispecific antibodies that engage NK cells by simultaneous binding to NKp46 and CD16 on NK cells and to a specific antigen on cancer cells show higher potency than current clinically available therapeutic antibodies generated against the same cancer antigen [21]. The modular nature of the ROCK^®^ platform has a built-in capability to generate trispecific ICE^®^ molecules that would harbor domains specific to two different NK cell receptors, potentially enabling them to recruit NK cells more efficiently. Moreover, this study provides strong evidence that bispecific ICE^®^ engaging solely the CD16A receptor is highly effective in mediating tumor cell lysis by NK cells and, when pre-complexed with NK cells, preserves full activity after a freeze and thaw cycle. We have previously shown that ICE^®^/tumor-antigen associated cross-link-experienced healthy donor-derived NK cells are triggered to phenotypic changes boosting proliferation and cytotoxic capacity in response cytokines, such as IL-2 and IL-15 [46]. Therefore, pre-complexing of ICE^®^ with CD16A on NK cells promotes additional benefits beyond targeting and increasing the ADCC-based cytotoxicity.

CAR-like NK cell products may show promise as effective therapeutic agents against minimal residual disease (MRD) in patients with different tumor types, in particular those with hematological malignancies. A recently published Phase 1/2 study investigated CD19-directed CAR-NK cells in 11 heavily pretreated patients with lymphoma and reported overall response rate of 73% (8/11 patients), with 88% of patients still being negative for MRD after 30 days from infusions at all dose levels [15]. CAR-like NK-cell products may also be effective in a broader population of patients with solid tumors, even when expression of a target antigen is low on the surface of cancer cells, and when patient NK cells have compromised ability to traffic, and recognize and attack tumor cells, due to the immunosuppressive tumor microenvironment or different lines of therapies that patients with cancer usually receive.

CAR-like NK-cell products may also achieve higher efficacy when used in combination with other immunotherapies, including ADCC-inducing IgG1, checkpoint inhibitors, or costimulatory molecules. This is supported by a recent study that showed the effectiveness of the combination of AFM13 (CD30/CD16A ICE^®^) and pembrolizumab in patients with R/R HL [47].

## 5. Conclusions

This study provides evidence of the superior efficacy of the ICE^®^ construct engaging the CD16A receptor in inducing ADCC when compared with the efficacy of ICE^®^ constructs specific for the NKp46 and NKG2D receptors. The magnitude and specificity of ADCC induced by the ICE^®^ and NK cell complexes remain unaffected after a single freeze–thaw cycle. These results suggest that CD16A-specific ICE^®^ constructs pre-complexed with NK cells may be amenable to further development as allogeneic off-the-shelf NK cell products.

## Figures and Tables

**Table 1:** The monovalent and multivalent binding affinities of bispecific ICE^®^ constructs to recombinant human EpCAM, CD16A, NKG2D, and NKp46 antigens.

Constructs	Interaction	K_D_ [nM]				
		EpCAM	CD16A^158V^	CD16A^158F^	NKp46	NKG2D
EpCAM/CD16A	monovalent	38.8 ± 5.6	13.9 ± 5.8	27.6 ± 10.1	n.d.	n.d.
	multivalent	0.7 ± 0.02	1.0 ± 0.05	1.2 ± 0.12	n.d.	n.d.
EpCAM/NKp46	monovalent	36.7 ± 5.5	n.d.	n.d.	18.0 ± 1.3	n.d.
	multivalent	0.4 ± 0.02	n.d.	n.d.	0.7 ± 0.01	n.d.
EpCAM/NKG2D	monovalent	68.2 ± 22.6	n.d.	n.d.	n.d.	53.6 ± 4.6
	multivalent	0.6 ± 0.03	n.d.	n.d.	n.d.	1.7 ± 0.2
Anti-EpCAM IgG1	monovalent	n.t.	n.t.	n.t.	n.t.	n.t.
	multivalent	0.3 ± 0.004	8.2 ± 0.4	8.5 ± n.a.	n.d.	n.d.
Anti-EpCAM Fc-enhanced IgG1	monovalent	n.t.	n.t.	n.t.	n.t.	n.t.
	multivalent	0.3 ± 0.04	5.9 ± 0.8	20.7 ± 3.0	n.d.	n.d.

The binding interaction of EpCAM/CD16A, EpCAM/NKp46, EpCAM/NKG2D, anti-EpCAM IgG1, and anti-EpCAM Fc-enhanced IgG1 was measured using SPR at 37 °C and an experimental set-up allowing for the monovalent or multivalent interaction (*n* = 3). Binding curves were fitted to a 1:1 binding model to evaluate equilibrium dissociation constants K_D_, and the arithmetic means ± standard deviation were calculated. n.d., not detectable; n.t., not tested; SPR, surface plasmon resonance.

**Table 2:** Binding affinity and specificity of ICE^®^ constructs to recombinant human EpCAM, CD16A, CD16B, NKG2D, and NKp46 antigens measured by ELISA.

Construct	Mean Values of EC_50_ [nM] ± SD					
	CD16A^158V^	CD16A^158F^	CD16B^NA1^	EpCAM	NKp46	NKG2D
EpCAM/NKp46	n.d.	n.d.	n.d.	0.9 ± 0.2	0.7 ± 0.1	n.d.
EpCAM/NKG2D	n.d.	n.d.	n.d.	1.2 ± 0.0	n.d.	0.6 ± 0.1
EpCAM/CD16A	1.1 ± 0.1	1.0 ± 0.0	n.d.	1.5 ± 0.0	n.d.	n.d.
Anti-EpCAM IgG1	42.1 ± 2.4	325.9 ± 154.0	n.d.	1.1 ± 0.0	n.d.	n.d.
Anti-EpCAM Fc-enhanced IgG1	5.2 ± 0.7	10.0 ± 2.0	251.7 ± 189.8	1.0 ± 0.0	n.d.	n.d.

EC_50_ are the mean values of two or three independent ELISA experiments ± standard deviation (SD). EC_50_, half-maximal binding constants; ELISA, enzyme-linked immunosorbent assay; n.d., not detectable.

**Table 3:** Apparent affinities of bispecific ICE^®^ constructs for CHO cells expressing recombinant human CD16A^158F^, CD16A^158V^, CD16B^NA1^, NKp46, and NKG2D.

Construct	K_D_ [nM] CD16A^158F^	K_D_ [nM] CD16A^158V^	K_D_ [nM] CD16B^NA1^	K_D_ [nM] NKp46	K_D_ [nM] NKG2D
EpCAM/NKp46	n.d.	n.d.	n.d.	1.7 ± 0.6	n.d.
EpCAM/NKG2D	n.d.	n.d.	n.d.	n.d.	0.6 ± 0.3
EpCAM/CD16A	27.7 ± 2.0	22.7 ± 10.5	n.d.	n.d.	n.d.
Anti-EpCAM IgG1	889 ± 396	308 ± 152	2692 ± 1928	n.d.	n.d.
Anti-EpCAM Fc-enhanced IgG1	80.7 ± 40.8	26.3 ± 15.8	176 ± 71	n.d.	n.d.
CD30/CD16A	24.9 ± 3.2	22.5 ± 9.9	n.d.	n.d.	n.d.

Bispecific ICE^®^ constructs and control antibodies were titrated on the CHO cell pools expressing the indicated recombinant antigens at 37 °C, and the cell-surface bound antibody constructs were detected with FITC-conjugated goat anti-human IgG Fc and flow cytometric analysis. MFI were used for the calculation of apparent affinities (K_D_) by non-linear regression. Mean K_D_ values derived from three independent experiments ± SD are shown. CHO, Chinese hamster ovary cells; FITC, fluorescein isothiocyanate; MFI, median fluorescence intensities; n.d., not detectable; SD, standard deviation.

**Table 4:** Apparent affinities of bispecific ICE^®^ constructs and control antibodies on enriched primary human NK cells measured by flow cytometry.

Construct	K_D_ [nM]
EpCAM/NKp46	0.5 ± 0.2
EpCAM/NKG2D	0.2 ± 0.1
EpCAM/CD16A	7.9 ± 0.7
Anti-EpCAM IgG1	1292 ± 766
Anti-EpCAM Fc-enhanced IgG1	55.5 ± 16.4
CD30/CD16A	6.7 ± 1.5

Bispecific ICE^®^ constructs and control antibodies were titrated on enriched primary human NK cells at 37 °C, and the cell-surface bound antibody constructs were detected with FITC-conjugated goat anti-human IgG Fc and flow cytometric analysis. MFI were used for the calculation of apparent affinities (K_D_) by non-linear regression. Mean K_D_ values derived from three independent experiments ± standard deviation are presented. FITC, fluorescein isothiocyanate; MFI, median fluorescence intensities.

**Table 5:** Apparent affinities of bispecific ICE^®^ constructs and control antibodies to EpCAM^+^ tumor cell lines.

Construct	K_D_ [nM] HCC-1954	K_D_ [nM] Detroit 562	K_D_ [nM] HCC-1187
EpCAM/NKp46	1.6 ± 0.7	1.6 ± 0.3	1.5 ± 0.7
EpCAM/NKG2D	1.7 ± 0.7	1.6 ± 0.3	1.5 ± 0.7
EpCAM/CD16A	1.7 ± 0.6	2.0 ± 0.3	1.9 ± 1.0
Anti-EpCAM IgG1	1.6 ± 0.6	1.5 ± 0.4	1.4 ± 0.7
Anti-EpCAM Fc-enhanced IgG1	1.3 ± 0.6	1.2 ± 0.2	1.2 ± 0.6
CD30/CD16A	n.d.	n.d.	n.d.

Bispecific ICE^®^ constructs and control antibodies were titrated on the indicated tumor cell lines at 37 °C, and the cell-surface bound antibody constructs were detected with FITC-conjugated goat anti-human IgG Fc and flow cytometric analysis. MFI were used for the calculation of apparent affinities (K_D_) by non-linear regression. Mean K_D_ values derived from three independent experiments ± standard deviation are presented. MFI, median fluorescence intensities; n.d., not detectable.

**Table 6:** Potency and efficacy of bispecific ICE^®^ constructs and control antibodies determined in NK cell fratricide assays.

Construct	EC_50_ [pM]	E_max_ [%]
EpCAM/NKp46	n.a.	0.0 ± 0.0
EpCAM/NKG2D	n.a.	0.0 ± 0.0
EpCAM/CD16A	2797 ± 1209	11.8 ± 3.8
CD30/CD16A	20893 ± 23747	21.7 ± 14.8
Anti-EpCAM IgG1	n.a.	13.1 ± 7.5
Anti-EpCAM Fc-enhanced IgG1	43242 ± 70322	9.0 ± 4.4
Anti-CD38 IgG1	73.6 ± 26.7	61.1 ± 6.1

Potency (EC_50_) and efficacy (E_max_) of the indicated ICE^®^ constructs and control antibodies were determined in three independent NK cell fratricide assays. Mean values ± standard deviation are shown. n.a., not applicable.

**Table 7:** Cytotoxicity of bispecific ICE^®^ constructs and control antibodies tested on target tumor cells with or without EpCAM and CD30 expression.

Construct	EpCAM^−^/CD30^+^ KARPAS-299				EpCAM^+^/CD30^−^ HCC-1954				EpCAM^+^/CD30^−^ Detroit 562				EpCAM^+^/CD30^−^ HCC-1187			
	EC_50_ [pM]		E_max_ [%]		EC_50_ [pM]		E_max_ [%]		EC_50_ [pM]		E_max_ [%]		EC_50_ [pM]		E_max_ [%]	
	Mean	SD	Mean	SD	Mean	SD	Mean	SD	Mean	SD	Mean	SD	Mean	SD	Mean	SD
EpCAM/NKp46	13994.0	n.a.	0.4	0.6	2.8	0.5	54.9	10.4	2.2	0.7	59.8	11.3	6.6	3.5	86.4	15.7
EpCAM/NKG2D	n.a.	n.a.	0.0	0.0	n.a.	n.a.	n.a.	n.a.	n.a.	n.a.	n.a.	n.a.	n.a.	n.a.	n.a.	n.a.
EpCAM/CD16A	n.a.	n.a.	0.0	0.0	2.7	0.7	64.6	5.4	1.6	0.4	70.6	12.0	2.6	1.3	101.5	9.8
Anti-EpCAM IgG1	20717.0	n.a.	3.2	2.8	11.6	5.1	73.5	9.0	6.6	3.3	72.6	12.0	40.1	35.9	98.7	13.2
Anti-EpCAM Fc-enhanced IgG1	5487.0	n.a.	1.8	3.1	3.2	0.6	69.0	8.3	1.6	0.3	69.2	12.8	6.6	4.7	100.9	6.2
CD30/CD16A	36.5	23.0	63.3	9.7	n.a.	n.a.	0.0	0.0	n.a.	n.a.	0.0	0.0	n.a.	n.a.	0.0	0.0

Potency (EC_50_) and efficacy (E_max_) were determined in 4 h calcein-release cytotoxicity assays on different target cells using enriched primary human NK cells as effector cells at an E:T ratio of 5:1. Mean values of three independent experiments ± standard deviation are shown. E:T, effector-cell-to-target-cell ratio; n.a., not applicable.

**Table 8:** Efficacy of cryopreserved and revived ICE^®^ constructs pre-complexed with NK cells.

ICE^®^ Construct-NK Cell Complexes	Efficacy [%] of Target Cell Lysis at an E:T Ratio of 5:1	
	HCC-1187 Mean ± SD	KARPAS-299 Mean ± SD
EpCAM/NKp46	46.1 ± 11.2	5.3 ± 4.0
EpCAM/NKG2D	14.3 ± 9.1	3.7 ± 5.5
EpCAM/CD16A	74.8 ± 15.3	3.1 ± 7.4
Anti-EpCAM IgG1	39.2 ± 31.3	5.3 ± 2.5
Anti-EpCAM Fc-enhanced IgG1	81.8 ± 8.3	4.8 ± 4.2
CD30/CD16A	0.6 ± 5.3	35.7 ± 19.3
w/o antibody	3.5 ± 3.9	5.9 ± 3.2
w/o antibody + EpCAM/NKp46	57.4 ± 10.0	4.2 ± 2.5
w/o antibody + EpCAM/NKG2D	23.6 ± 1.3	3.1 ± 3.1
w/o antibody + EpCAM/CD16A	83.3 ± 5.0	4.8 ± 3.9
w/o antibody + CD30/CD16A	n.t.	52.1 ± 2.9 *

* Mean and SD from two independent experiments. NK cells were pre-complexed with or without 10 µg/mL of the indicated ICE^®^ constructs and control antibodies for 30 min at room temperature and then frozen at −80 °C. Thawed NK cells were washed and used as effector cells in a 4 h calcein release cytotoxicity assay against HCC-1187 or KARPAS-299 target cells. As a control, fresh EpCAM/NKp46, EpCAM/NKG2D, EpCAM/CD16A, and CD30/CD16A ICE^®^ constructs were added at a concentration of 10 µg/mL to NK cells that have not been pre-complexed prior to freezing (w/o antibody). Mean ± SD of lysis values at an E:T ratio of 5:1 from three experiments are shown. E:T, effector-cell-to-target-cell ratio; n.t., not tested; SD, standard deviation; w/o, without.

## Data Availability

Data are contained within the article or supplementary material.

## Supplementary Materials

The following supporting information can be downloaded at: https://www.mdpi.com/article/10.3390/antib11010012/s1, Figure S1: Thermal stability of bispecific ICE® constructs evaluated by DSF. The melting profile of ICE® constructs was assessed by recording the fluorescence signal (change of fluorescence signal over change of temperature dF/dT) after an increase in temperature by 1 Kelvin/minute and addition of SYPRO® Orange. dF/dT, fluorescence intensity/temperature; DSF, differential scanning fluorometry, Table S1: Design of bispecific ICE® constructs, Table S2: Thermal stability of bispecific ICE® constructs evaluated by DSF, Table S3: ICE® construct stability, Table S4: Cell-surface EpCAM expression levels on tumor cell lines, Table S5: Retention of ICE® constructs and control antibodies on the surface of NK cells at different dissociation phases.
