# Supplementary material for: Cryopreservation of Natural Killer Cells Pre-Complexed with Innate Cell Engagers
Source: Antibodies (Basel). 2022 Feb 9;11(1):12. doi: 10.3390/antib11010012 (PMC8883965; doi:10.3390/antib11010012)
Supplement: Supplementary file 1 [file antibodies-11-00012-s001.zip › antibodies-1540739-supplementary.pdf]

Supplementary

Cryopreservation of Natural Killer Cells Pre-complexed with Innate Cell Engagers

Table S1. Design of bispecific ICE® constructs.

Bispecific ICE® constructs	Format	Target specificity	Target domain	Position of targeting domain	Effector specificity	Effector domain	Position of effector domain
EpCAM/NKp46	scFv-IgAb	EpCAM	42	Fab	NKp46	NKp46-1	scFv
EpCAM/NKG2D	scFv-IgAb	EpCAM	42	Fab	NKG2D	KYK_2_0	scFv
EpCAM/CD16A	scFv-IgAb	EpCAM	42	Fab	CD16A	P2C-47	scFv
CD30/CD16A	scFv-IgAb	CD30	HRS3	Fab	CD16A	P2C-47	scFv
Anti-EpCAM IgG1	IgG	EpCAM	42	Fab	n.a.	n.a.	n.a.
Anti-EpCAM Fc-enhanced IgG1	IgG	EpCAM	42	Fab	n.a.	n.a.	n.a.

Fab, antigen-binding fragment; n.a., not applicable; scFv, single-chain variable fragment.

Table S2. Thermal stability of bispecific ICE® constructs evaluated by DSF.

Construct	Additional Tm [°C]	Tm1 [°C]	Tm2 [°C]
EpCAM/NKp46	54.0	62.5	73.2
EpCAM/NKG2D	n.a.	61.7	72.5
EpCAM/CD16A	n.a.	62.0	71.5
Anti-EpCAM IgG1	n.a.	63.5	75.0
Anti-EpCAM Fc-enhanced IgG1	n.a.	47.5	74.3

The melting profile of ICE® constructs was assessed by recording the fluorescence signal following an increase in temperature by 1 Kelvin/minute and addition of SYPRO® Orange. DSF, differential scanning fluorometry; Tm, transition midpoint temperatures. n.a., not applicable

Table S3. ICE® construct stability

Construct	Relative product content [%]					
		4–8°C	25°C	40°C	−80°C	pH 3.5
	0 days	7 days	7 days	7 days	3 freeze/thaw cycles	90 min
EpCAM/NKp46	97.8	97.4	97.7	96.4	98.0	98.0
Ep-CAM/NKG2D	97.7	96.7	97.3	97.3	97.7	95.6
EpCAM/CD16A	98.2	98.4	97.9	94.9	97.3	98.1
Anti-Ep-CAM Fc-en-hanced IgG1	96.4	95.6	95.7	94.1	95.5	96.0

ICE® constructs used at 1.5 mg/mL concentration were either incubated at 4–8°C, 25°C, or 40°C for 7 days, subjected to three freeze/thaw cycles at −80°C/ambient temperature or incubated at pH 3.5 for 90 min. The ICE® construct stability was measured by SE-HPLC. SE-HPLC, size exclusion-high-performance liquid chromatography; no data available for the CD30/CD16A ICE and anti-EpCAM IgG1.

Table S4. Cell-surface EpCAM expression levels on tumor cell lines

Cell line	Mean SABC ± SD
Detroit 562	215240 ± 50255
HCC-1954	184253 ± 5988
HCC-1187	111931 ± 21910

SABC per cell was determined by calibrated flow cytometry using anti-EpCAM mAb 323/A3. Mean values ± standard deviation of three independent assays are shown. mAb, monoclonal antibody; SABC, specific antibody binding capacity.

Table S5. Retention of ICE® constructs and control antibodies on the surface of NK cells at different dissociation phases

Construct	Relative amount of remaining antibodies after dissociation [%]		
	6 hours Mean ± SD	24 hours Mean ± SD	48 hours Mean ± SD
EpCAM/NKp46	52.0 ± 11.6	15.9 ± 16	9.0 ± 0.1
EpCAM/NKG2D	4.9 ± n.a.	2.2 ± n.a.	n.a.
EpCAM/CD16A	44.3 ± 9.3	17.3 ± 13.3	8.2 ± 12.3
Anti-EpCAM IgG1	−2.7 ± 2.2	−3.0 ± 2.1	n.t.
Anti-EpCAM Fc-enhanced IgG1	−1.0 ± 1.2	−1.0 ± 1.2	n.t.
CD30/CD16A	48.4 ± 9.8	21.6 ± 12.7	10.4 ± 12.5

Aliquots of enriched primary human NK cells were pre-complexed with 100 μg/mL of the indicated antibody constructs on ICE®, washed, and then incubated at 37°C in a large volume of medium for various time periods to allow dissociation. The retained antibodies were detected by FITC-conjugated goat anti-human IgG and the flow cytometric analysis. MFI determined for each antibody at the time point 0 was set to 100%, MFI of NK cells stained with secondary reagent alone was set to 0, and the relative amount of retained antibodies was calculated. MFI for some samples were lower than MFI for staining with a secondary reagent alone, leading to negative values; values <-10% were excluded. Mean values ± standard deviation from three independent experiments are shown. FITC, fluorescein isothiocyanate; ICE®, innate cell MFI, median fluorescence intensity; n.a., not applicable; n.t., not tested.

Figure S1. Thermal stability of bispecific ICE® constructs evaluated by DSF

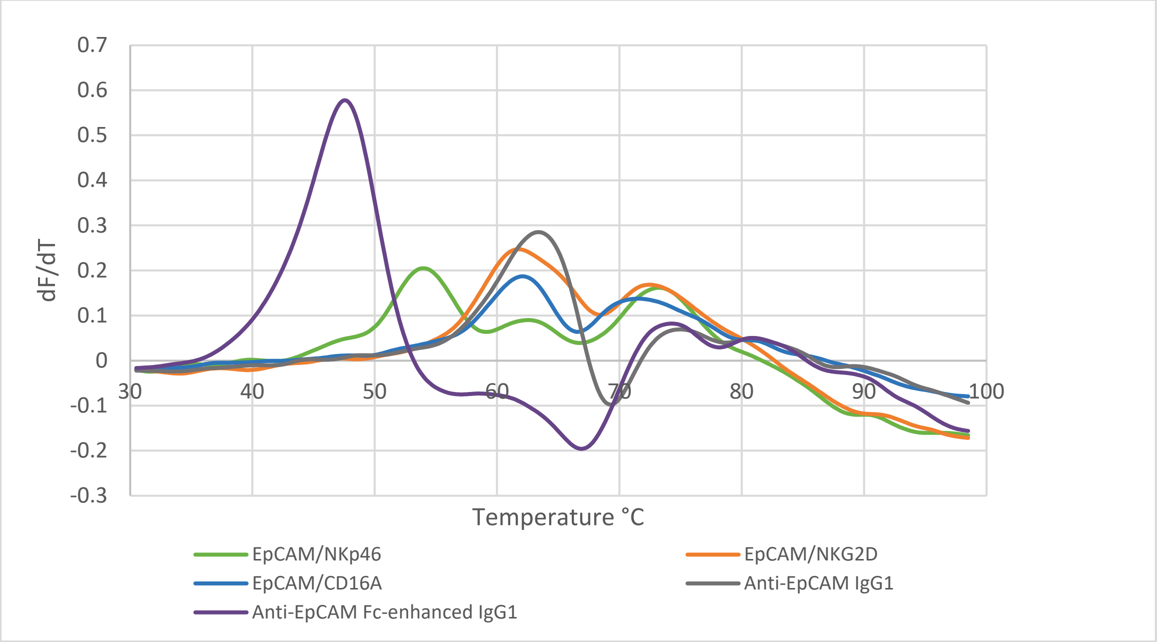

The melting profile of ICE® constructs was assessed by recording the fluorescence signal (change of fluorescence signal over change of temperature dF/dT) after an increase in temperature by 1 Kelvin/minute and addition of SYPRO® Orange. dF/dT, fluorescence intensity/temperature; DSF, differential scanning fluorometry.
